# Laparoscopic extraperitoneal para-aortic lymphadenectomy

**DOI:** 10.3332/ecancer.2015.573

**Published:** 2015-09-21

**Authors:** Pablo Padilla Iserte, Lucas Minig, Cristina Zorrero

**Affiliations:** Valencian Institute of Oncology (IVO), Department of Gynecology, Clinical Area of Gynecologic Oncology, Profesor Beltran Baguena, 8, Valencia 46009, Spain

**Keywords:** para-aortic lymph nodes, retroperitoneum space, laparoscopy, surgical staging, endometrial cancer, ovarian cancer

## Abstract

Lymph nodes are the main pathway in the spread of gynaecological malignancies, being a well-known prognostic factor. Lymph node dissection is a complex surgical procedure and requires surgical expertise to perform the procedure, thereby minimising complications. In addition, lymphadenectomy has value in the diagnosis, prognosis, and treatment of patients with gynaecologic cancer. Therefore, a video focused on the para-aortic retroperitoneal anatomy and the surgical technique of the extraperitoneal para-aortic lymphadenectomy is presented.

## Introduction

Aortic lymphadenectomy can be performed for therapeutic and staging purposes in women affected by gynaecologic malignancies. A well-defined boundary of dissection with a sufficient number of removed nodes is required to perform a surgical staging with maximum therapeutic benefit [1].

Lymphadenectomy in gynaecologic cancer has been traditionally performed by laparotomy. However, in recent decades, the laparoscopic approach has been associated with fewer postoperative complications including less blood loss, minor postoperative adhesion formation, and shorter hospital stay than laparotomy, with an equivalent assessment of lymph node status [2].

Laparoscopic para-aortic lymph node dissection can be performed through the extraperitoneal or transperitoneal approach. Retroperitoneal para-aortic lymphadenectomy was described by Dargent in 2000 [3]. Recent evidence has demonstrated that the extraperitoneal approach is associated with a higher number of lymph nodes removed with similar surgical outcomes as the transperitoneal route [4].

Therefore, a surgical film focused on the anatomy of para-aortic retroperitoneal spaces and the surgical technique of extraperitoneal para-aortic lymphadenectomy is presented (Video 1).

## Clinical case

A 67-year-old, otherwise healthy woman, G3 P3 with BMI of 23.4 kg/m^2^, presented with postmenopausal bleeding. Pelvic examination showed an atrophic cervix without lesions, with an enlarged uterus reaching navel level, without involvement of parametria and vagina walls. The Eastern Cooperative Oncology Group (ECOG) performance was 0.

**Figure 1.**
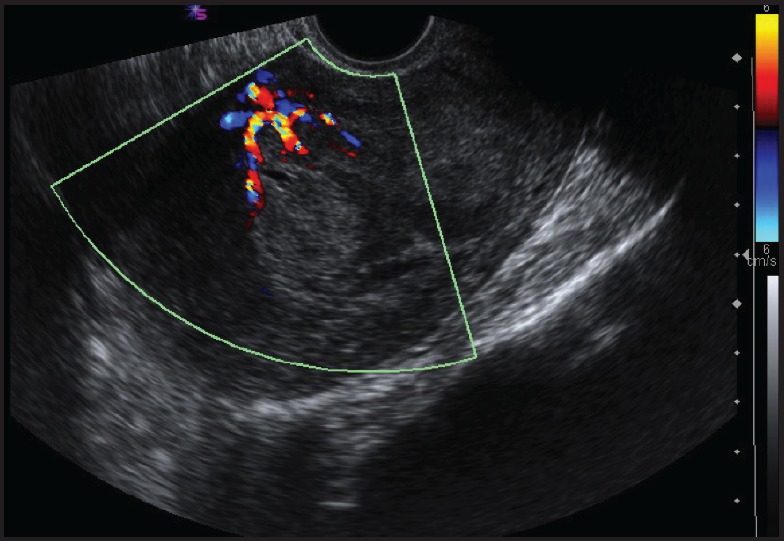
Transvaginal ultrasound described an enlarged uterus with endometrial thickening, confined to endometrial cavity. Important vascularisation detail, by applying colour Doppler.

A transvaginal ultrasound showed an enlarged uterus with heterogeneous endometrial thickening, with a myometrial invasion higher than 50%, limited to the endometrial cavity (Figure 1).

**Figure 2.**
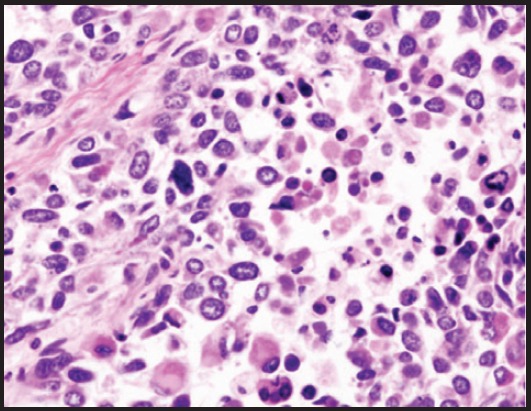
Pathological study showed a large cells with eosinophilic cytoplasm, pleomorphic nuclei with irregular nucleoli.

A hysteroscopy-guided biopsy revealed uterine carcinosarcoma (Figure 2). A chest and abdominopelvic computed tomography (CT) did not show extrauterine disease, nor nodal metastasis. Surgical staging was decided at the institutional tumour board. The surgery started with performing a laparoscopic extraperitoneal para-aortic lymphadenectomy, followed by hysterectomy with bilateral salpingo-oophorectomy and transperitoneal pelvic lymphadenectomy. No evidence of extra-uterine macroscopic disease was noted during surgery.

The surgical specimen confirmed an endometrial carcinosarcoma with myometrial invasion higher than 50%, without serosal invasion. Pelvic lymphadenectomy revealed 19 nodes without neoplastic infiltration. A total of 14 out of 21 para-aortic nodes removed had macrometasis, two of them with extracapsular spread.

A uterine carcinosarcoma, International Federation of Gynaecology and Obstetrics (FIGO) stage IIIC_2_ was diagnosed. The patient received adjuvant chemotherapy based on six cycles of intravenous carboplatin and paclitaxel.

## Extraperitoneal para-aortic lymphadenectomy: description of the technique

The patient is placed in the dorsal decubitus position, close to the left margin of the surgical table. The right arm is extended and fixed while the left arm is abducted 90º. The surgeon is placed to the left side of the patient and the assistant is to the left of the surgeon. The monitor is placed in front of them. The extraperitoneal para-aortic lymphadenectomy starts with performing an inspection of the abdominal cavity through a 10-mm umbilical trocar. At this time, the presence and extension of intra-abdominal disease can be observed while peritoneal lavage is being performed [5].

Next, a 10-mm incision is made 3 to 4 cm medially to the left anterior iliac spine in the midclavicular line, using an extraperitoneal approach. The surgeon uses the index finger to carefully open the space between the peritoneum and muscles of the abdominal wall under transperitoneal laparoscopic visualisation to prevent peritoneum rupture, as showed in the video. The surgeon should detect the pulsation of the external iliac vessels along the psoas muscle. It should complete the digital dissection laterally and cranially along the left psoas [3].

**Figure 3.**
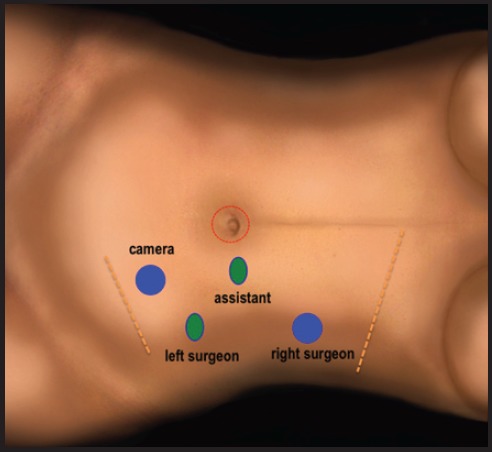
Trocars position in the left side to access extraperitoneal space (in blue 10-mm trocars, in green 5-mm trocars, and red one for transperitoneal access).

**Figure 4.**
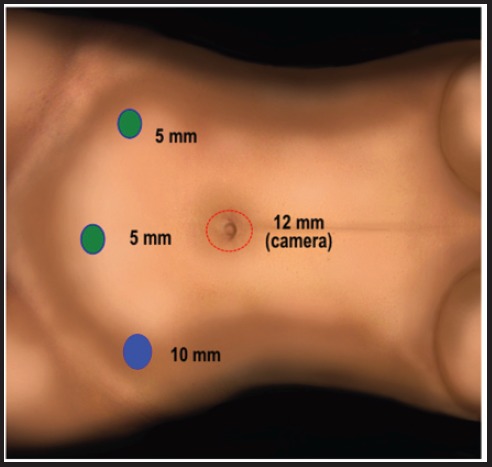
Trocars position in the transperitoneal space (in blue 10-mm trocars and in green 5-mm trocars).

Once the extraperitoneal space has been prepared, a 10-mm trocar is introduced, CO_2_ is insufflated, and the peritoneal cavity is simultaneously emptied in order to avoid pressure over the extraperitoneal space. Similar to the intra-abdominal space, the insufflation’s pressure is 10 mm–12 mm Hg; flow 3 L/minute. Additional 10-mm and 5-mm trocars are placed along the anterior axillary line at the subcostal margin, above the iliac spine (Figure 3–4).

## Retroperitoneal anatomy and dissection planes

The aortic region includes the area from renal vessels to the aortic bifurcation. The anatomical dissection of the retroperitoneal spaces is the first step in the para-aortic lymphadenectomy. It is important to note that optimal exposures of the aorta and the vena cava from the bifurcation to the renal vessels are the most important factors to obtain successful surgical outcomes.

**Figure 5.**
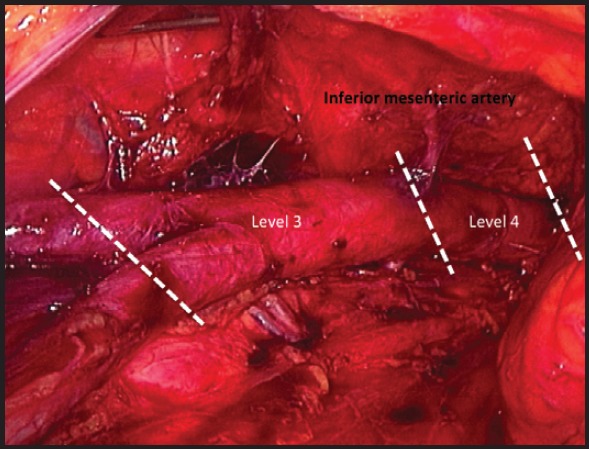
Retroperitoneal image: aortic region anatomy level 3 and 4 proposed by Querleu, separated by the inferior mesenteric artery (upper limit: renal veins; lower limit: aortic bifurcation).

The relationship between the lymphatic tissue and the great vessels may be divided in two anatomical compartments proposed by Querleu and Morrow [6]. Thus, the tissue extended from aortic bifurcation to the inferior mesenteric artery (level 3) and the area between the inferior mesenteric artery to the renal veins (level 4) is dissected (Figure 5). The level 1 and 2 correspond with pelvic anatomy. Lymph nodes on the vena cava are called precaval and paracaval to its right side. The nodes located between the aorta and the vena cava, are the intercavo-aortic ones. Finally, the tissues placed at the left side of the aorta are called para-aortic, while the pre-aortic ones are at the anterior side [1].

The left psoas muscle is medially dissected from the peritoneum. The left ureter is identified on the anterior surface of the psoas and is separated with the peritoneum, being the roof of the dissection plane. Nodal dissection starts at the aortic bifurcation and ends with the level of the renal veins (it is identified directly or by following the left gonadal vein). The peritoneal sac is elevated, allowing the identification of the sacral promontory and the bifurcation of the aorta and the inferior vena cava. The right common iliac vein and the right common iliac artery are dissected by gentle manoeuvres. The right ureter is identified and then elevated; next it is separated from the iliac vessels and right psoas muscle. Cranial dissection should continue with lymph dissection above the inferior vena cava.

Finally, lymph nodes are carefully removed by an Endobag through the 10-mm port. Haemostasis should be evaluated. Then, the extraperitoneal space is deflated and the abdominal cavity is insufflated, a 2-cm incision is made in the peritoneal sac in order to avoid lymphocyst formation.

## Discussion

The first laparoscopic para-aortic lymphadenectomy was made using the transperitoneal approach [7]. The main advantages of the minimally invasive approach over traditional laparotomy include: shorter hospital stay, less blood loss, faster recovery, less pain, smaller scar, and faster return to bowel function [2] with an equivalent assessment of lymph node status.

A minimally invasive para-aortic lymphadenectomy can be performed following two different routes: the transperitoneal or the extraperitoneal approach. The main limitations of the transperitoneal route include poor exposure of surgical field, difficulties with the mobilisation and retraction of the small bowel, appropriated identification of the ureters, and mobilisation of the sigmoid colon [8]. These limitations can be even worse in case of truncal obesity (BMI ≥ 35 kg/m^2^), short mesentery, distended large bowel, intestinal adhesions, or intolerance to Trendelemburg position. Under these circumstances, the procedure can be time consuming, highly difficult, and with high rates of intraoperative complications and conversion to laparotomy [4].

The extraperitoneal route arises as a valid alternative to reduce the limitations previously described, improving the access to the retroperitoneum in order to improve the surgical results with a simpler and safer approach [9]. The main advantages include a faster access to the lymph nodes area with an adequate exposure of the surgical field. Trendelenburg position is not necessary, thus reducing the patient’s haemodynamic instability during surgery. In addition to avoiding intra-abdominal procedures, the risk of postoperative complication, including ileus and intestinal obstruction, is significantly reduced [10].

Maintaining the integrity of the peritoneum is a key factor during the extraperitoneal para-aortic lymphadenectomy. In some cases, however, the peritoneum could be damaged and opened, losing the surgical field exposure advantage provided by the pneumoperitoneum. Possible solutions to this inconvenience include occluding the foramen with a Foley catheter balloon, or performing a closure with a running suture by using a transperitoneal approach [11]. In some circumstances the hole is impossible to close and the procedure needs to be converted to a transperitoneal route or directly to laparotomy.

Another inconvenience of the extraperitoneal approach is the lymphocele formation. Leblanc showed that postoperative lymphocele was present in 40 of the 104 first patients (rate of 13.4%). After doing a preventive marsupialisation of the left paracolic gutter, however, only three patients of 77 (3.8%) developed the complication [12].

Robotic-assisted para-aortic lymphadenectomy is another option to complete the procedure using the minimally invasive approach. This surgical system provides a steady three-dimensional view and improves the surgeon's movements (tremor filtration) with more precision, accuracy, and security [8]. Preliminary results may offer advantages over standard laparoscopy [13, 14], but one of the main limitations is the cost of the procedure. Further prospective clinical trials are necessary to better define the role of the retroperitoneal robotic assistance in the management of gynaecologic malignancies.

## Conclusions

The extraperitoneal approach is the first choice to perform a laparoscopic para-aortic lymphadenectomy. However, an adequate learning curve and an in-depth knowledge of the retroperitoneal anatomy are highly important for this approach.

## Figures and Tables

**Video 1:**
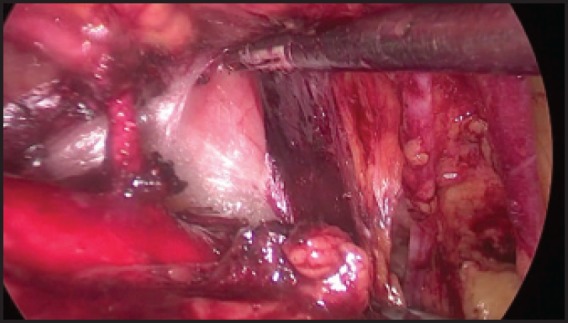
Extraperitoneal para-aortic lymphadenectomy. Video scene during the para-aortic dissection. To view this video click here: http://ecancer.org/journal/9/full/573-Laparoscopic-extraperitoneal-para-aortic-lymphadenectomy.php.
